# How Sensory Experiences Affect Adolescents with an Autistic Spectrum Condition within the Classroom

**DOI:** 10.1007/s10803-015-2693-1

**Published:** 2016-01-20

**Authors:** Fiona E. J. Howe, Steven D. Stagg

**Affiliations:** Department of Psychology, Anglia Ruskin University, Cambridge Campus, East Road, Cambridge, CB1 1PT UK

**Keywords:** Autism, Sensory processing, Adolescence, School

## Abstract

Sensory processing difficulties are consistently reported amongst individuals with an autistic spectrum condition (ASC); these have a significant impact on daily functioning. Evidence in this area comes from observer reports and first-hand accounts; both have limitations. The current study used the Adolescent/Adult Sensory Profile (AASP; Brown and Dunn in The Adolescent/Adult Sensory Profile: self questionnaire. Pearson, 2002a), and a qualitative questionnaire to investigate sensory issues in school children with ASC. The AASP found that the participants’ mean scores were outside normal parameters. Participants reported difficulties in at least one sensory domain, with hearing affecting them the most. Content analysis revealed sensory sensitivity to affect the participant’s learning and that sensory experiences were largely negative. Results suggest that schools need to create sensory profiles for each individual with ASC.

## Introduction

Sensory processing difficulties have been consistently reported amongst individuals with an autistic spectrum condition (ASC; e.g., Kern et al. 2006; Orekhova et al. 2008). Estimates of the prevalence of sensory features in ASC vary from 45 to 96 % (Ben-Sasson et al. 2007; Leekam et al. 2007; Tomchek and Dunn 2007). In ASCs, sensory difficulties are reported across ages (Kern et al. 2006), in both genders (Lai et al. 2011) and across severity (Leekam et al. 2007). Previous diagnostic systems neglected the importance of sensory processing issues in individuals with ASC (e.g., DSM-IV-TR; American Psychiatric Association; APA 2000), but recent updates have recognised that altered sensory processing is an experience many individuals with ASC report (DSM-5; APA 2013). While sensory difficulties are noted in the literature, it is rare to find self-reports documenting the sensory difficulties individuals experience and even less frequent are self-reports from school aged children. This paper presents a qualitative account of how a group of mainstream school children with ASC report their subjective experiences of sensory issues, and how they feel these issues impact on their school lives.

Sensory processing issues in ASC cover a broad spectrum from unisensory issues such as hyper/hypo sensitivity to specific stimuli through to multisensory issues that involve integrating information from different senses. For example, individuals with ASC have difficulties with multisensory integration and may fail to bind co-occurring stimuli (e.g., lip movement and phoneme sound) resulting in social communication problems or the failure to use combined social cues from facial expression and voice tone (Kwakye et al. 2011). While multisensory binding may have a development impact on the individual, other sensory abnormalities are more immediate subjective experiences and may include painful reactions to every day sensory stimuli such as light and sound. Within the unisensory domain, there are three classifications of sensory difficulties that are experienced by people with ASC: sensory sensitive, sensory insensitive (Watling et al. 2001) and sensory seeking (Miller et al. 2007). The classification experienced is dependent on both the situation and the sensory modality (Baranek et al. 2006).

Hyper and hypo sensory issues can have a major impact on the life of the individual experiencing sensory disturbance. Families with children with ASC adapt their daily lives around the sensory issues of their children, and sensory processing issues often exclude their children from joining in certain activities (Schaaf and Zoghbi 2011). While sensory issues may be easier to accommodate in the home environment, the school environment presents particular challenges that may heighten sensory issues in the child (Fernández-Andrés et al. 2015). For example, Humphrey and Lewis (2008) documented anxiety in children with ASC caused by having to move through corridors full of people pushing into each other. Accessing school is part of a child’s everyday life, but for a child with ASC this comes with multiple challenges (Humphrey and Lewis 2008). Brown and Dunn (2010) explored differences in sensation seeking and avoiding in children with ASC when they were at home and when they were at school. These two quadrants were found to have good and fair correlations, which led to the conclusion that these sensory processing styles have qualities that are both universal and context specific. For example, with sensory avoiding, both the teacher and the parent may report a child to put their hands over their ears in response to a noise; however, if the child’s home environment is quieter than their school environment, the parent would observe fewer auditory reactions than the teacher.

Inability to concentrate or over preoccupation with sensory stimuli may have negative outcomes for the child’s schooling. For example, Ashburner et al. (2008) compared the sensory processing patterns and educational outcomes of children with ASC to neurotypical children with matched intelligence quotients. The results showed that for the children with ASC, difficulties with auditory filtering, sensory under-responsivity and sensory seeking were associated with underachieving academically. Hilton et al. (2010) found atypical sensory responses to be possible predictors of the severity of social impairment and that the more severe a participant’s sensory issues were the more social problems they experienced. This would mean sensory processing issues also have an impact on the child’s ability to socialise with classmates.

In order to better understand the sensory experiences of children in the classroom research needs to access the first—hand accounts of these children. However, the majority of data in this area is collected using third hand reports (e.g., Tomchek and Dunn 2007), and there are a limited amount of self-report and autobiographical accounts that provide a subjective view of people with ASC living with these sensory processing difficulties. Research conducted on children most commonly uses the Sensory Profile (SP; Dunn 1999) to gain information about processing abnormalities. The SP is designed specifically for children aged 3–10 years of age, and is completed by a caregiver who has daily contact with the child. Relying on data collected from an observer, specifically one who is a care-giver, is a limitation due to individual judgment differences and emotional bias (Reynolds and Lane 2008). First-hand reports describing sensory difficulties are less well documented, but those that do exist provide an insight into individual experiences. In adults, sensory processing issues have been described as fear provoking (e.g., Volkmar and Cohen 1989) and causing physical pain (e.g., Smith and Sharp 2013). Research investigating the school environment is sparse with information coming from autobiographical accounts (Grandin 2014) and few research studies. Ashburner et al. (2013) employed qualitative techniques to study this issue, and interviewed three adolescents in research which begins to uncover the subjective experiences of sensory processing issues. Her work was able to highlight (on a relatively small sample) how sensory pain is experienced and what measures individuals take to avoid potentially stressful sensory situations. In order to gain an in depth explanation of the real life experiences of individuals with ASC, more qualitative research is needed in the area of special education needs and specifically autism (DfES 2001; Tavassoli et al. 2013; Prince-Hughes 2002). This can then be combined with scientific theories and empirical research to create a more detailed understanding of this disability (Chamak et al. 2008).

The current study investigates the experiences of children with ASC whilst they are in a classroom at school and does so using a qualitative technique in order to access the children’s subjective experiences of sensory issues within this environment. We also used the Adolescent/Adult Sensory Profile (AASP; Brown and Dunn 2002a) to establish objectively whether or not our sample did in fact represent a group of ASC children with sensory processing issues. The specific research question for the qualitative section is ‘How do adolescents with autistic spectrum conditions perceive sensory differences to be affecting their learning experiences within the classroom?’

## Methods

### Design

The qualitative data was collected through the use of a specifically designed questionnaire that required written responses. Some individuals with ASC prefer to communicate through written rather than verbal responses, and they feel more able to express themselves in this form than in direct social situations (Jones et al. 2003). Whilst vocalising feelings can be difficult for a person with ASC, written language can come quite fluently (Attwood 1998; Gerland 2003). Quantitative data was collected using the AASP.

### Participants

Adolescents who had a diagnosis of ASC and were currently attending school were approached. The participants all attended mainstream school. Sixteen participants took part in the research (12 males and 4 females) aged between 12 and 17 (mean age of 14.4 years). All participants had an existing diagnosis of an ASC and had a Statement of Special Education Needs (SSEN) where ASC was stated as their primary learning difficulty. The participants did not have a diagnosis of any co-morbid learning disabilities, and their Special Educational Needs Co-ordinator (SENCo) verbally confirmed the child’s ability to comprehend the study. The participants came from three schools in the East of England.

#### School 1

Four male participants came from School 1. All attended the Enhance Resources Centre (EHC) at this school. In order to attend the EHC a child has to be working at National Curriculum level 2 and needs extra support for the mainstream curriculum but are too able for an Area Special School. The children are in the EHC for literacy and numeracy lessons, and are taught in small numbers with extra support. They attend mainstream for their other lessons with 1:1 full time support.

#### School 2

Nine participants (two female and seven male) came from School 2. This school has an ASC Centre that all of the participants attend. The aim of the provision is to provide a calm and structured learning environment that reduces anxiety levels and increases learning potential in pupils with autism. The pupils attend at least the beginning of mainstream lessons in order to receive the ‘teaching’ part of a lesson and can then go back to the provision to complete their work with a teaching assistant if they need to. The provision is purely for pupils with autism and they have to have a SSEN with autism identified as their primary need in order to attend. They also have to have the ability to access a mainstream classroom under the appropriate support.

#### School 3

Two female participants came from School 3. These participants attended mainstream classes and have access to support from TA’s where it is appropriate.

### Refusal and Drop Off Rates

Five secondary schools were contacted: four schools responded and three gave their permission for the study to take place. From these schools 25 children were approached by their Special Educational Needs Coordinator to take part in the study and 16 of the parents and children gave their consent. Six of the parents did not give consent because they thought the study would cause their children too much distress.

Out of the 16 participants that agreed to take part in the research 15 completed both questionnaires. One participant did not complete all of the sections in the AASP, but they did complete the qualitative questionnaire.

Informed consent was obtained from all individual participants included in the study. Full ethical approval was received by the Faculty Research Ethics Panel for Science and Technology at Anglia Ruskin University.

### Procedures and Data Collection Methods

No procedure was the same because the participants had the choice of whether they completed the study at home or at school, and had the option of support in the form of a teaching assistant or a parent. To avoid them becoming overwhelmed, they did not have to complete the study all at once. A total of six participants chose to complete the study at home and five completed it independently. All the participant’s responses were recorded in written format; those who had assistance would either have written the answers for themselves or dictated their answer for their support to write down.

#### Measurements: AASP (Brown and Dunn 2002a)

The AASP is a self-report questionnaire. It is designed to measure the effect of sensory processing on functional performance and to identify an individual’s sensory processing pattern. It consists of 60 items that are split into six sections: taste/smell processing, movement processing, visual processing, touch processing, activity level and auditory processing. Each item belongs to a quadrant: low registration, sensation seeking, sensory sensitive and sensation avoiding. The items describe behaviours the participants have to rate depending on how often they perform them. This rating is done on a Likert type scale from almost never to almost always. If the participants have never experienced a behaviour described they left this item blank. The internal consistencies of the quadrants are between 0.64 and 0.78 (Cronbach’s alpha). Reliability statistics for the quadrants are between 0.639 and 0.775 (alpha coefficients) and validity statistics are between 3.58 and 4.51 (standard errors of measurement; Brown and Dunn 2002b).

##### Questionnaire

The questionnaire was created by the researcher for the current research to collect data about the participant’s sensory experiences. It consisted of four sections, one for each sense: touch, hearing, vision and smell. The design of the questions was based on worksheets produced by Attwood (1998) and Gaus (2011). The questions were chosen to address the research question. This process started by designing a semi-structured interview and then altering the questions to make them suitable for written responses. Questions took the form of rating scales and open and closed questions that needed written answers. See “Appendix 1” for a full copy of the questionnaire.

### Qualitative Data Analysis

The technique used to analyse the data was content analysis (Marks and Yardley 2004); this involves the identification of codes within the data and counting the frequency in which they appear. The data display methods were a combination of Connor (2012) and Finlay and Lyons (2001). This method consists of displaying the analysis of frequency counts in a number of tables, making the codes and counts clearly seen and patterns across the data apparent. The analysis process began with the lead researcher familiarising herself with the data. Then the participants’ answers were separated into individual comments, and notes for initial codes were made. The codes began as open and flexible accounts to allow for development throughout the analysis with the codes becoming more final as the analysis progressed. Similar codes were amalgamated, and this was repeated until no further condensing was possible. Themes capturing the elements of each group of codes were attributed. Part of this process involved using exclusive coding; each piece of data belonged to only one code. This meant making sure that the code descriptions were clear enough for the data to be included in only that one code, and there were no possible overlaps with another. The aim of the code definitions was to describe the code in a way that meant another researcher could place the data in the exact same code.

Once the process of coding was complete an independent coder (not involved with this study) placed the data in the established codes and a test of inter-rater reliability was performed (Cohen’s Kappa = 0.90, *p* < .001) which suggested that the codes were clear and applicable. To ensure codes were accurately represented and to provide evidence of code consistency across participants, quotes were carefully selected to describe the codes and a full list is included in “Appendix 2”. The analysis used all of the data; deviant cases were put in *other* and were discussed if they related to other existing codes. Whilst this could be interpreted as ignoring data, content analysis specifically analyses themes and a single occurrence would not be sufficient. A selection of code examples were given for each code (see “Appendix 2” for the complete coding manual), so that as recommended by Marks and Yardley (2004), readers can make their own decision about coding quality. The comments that are in the code *other* have been presented in the appendices for the same reason (see “Appendix 3” for the *other* comments).

## Results

### The Adolescent/Adult Sensory Profile

The data from two participants was removed due to incorrect completion of the measure; in total the data from 14 participants was used. Some items on the sensory profile were left blank, and this resulted in <2 % of missing data. Little’s missing completely at random test was performed on the data set to assess whether the data were missing at random. The resulting Chi square analysis was not significant *x*^2^(462) < .0001, *p* = 1 supporting the hypothesis that the missing data conformed to a random pattern. Missing values were then replaced using the expectation–maximisation method in SPSS.

Table 1 shows the sensory profile classifications and average quadrant scores for each participant. Two participants scoring outside the normal range on one quadrant (14 %), four scored outside the normal range on two quadrants (29 %), six scored outside the normal range on three quadrants (43 %) and two scored outside the normal range on four quadrants (14 %). These data suggest that all participants experienced some sensory issues outside of normal parameters.

**Table 1:** The participant’s scores from the Adolescent/Adult Sensory Profile (Brown and Dunn 2002a)

Participant^a^	Classifications for sensory profile quadrants^b^				Number of quadrants affected
	Low registration	Sensation seeking	Sensory sensitivity	Sensation avoiding	
1	More than most	Less than most	More than most	More than most	4
2	More than most	Similar to most	More than most	More than most	3
3	More than most	Less than most	Much more than most	More than most	4
4	More than most	Similar to most	More than most	More than most	3
5	More than most	Similar to most	More than most	More than most	3
6	More than most	Similar to most	More than most	Similar to most	2
7	Similar to most	Similar to most	Less than most	Similar to most	1
8	More than most	Less than most	Similar to most	Similar to most	2
9	Similar to most	Less than most	Similar to most	Similar to most	1
10	Similar to most	Less than most	Much more than most	Similar to most	2
11	More than most	Less than most	More than most	Similar to most	3
12	More than most	Similar to most	More than most	More than most	3
13	Much more most	Similar to most	Much more than most	More than most	3
14	Much more most	Similar to most	More than most	Similar to most	2
Percentage of participants scoring outside the normal range	79	43	93	50	
Average quadrant scores, ranges and standard deviations	*M* = 43 (26–57)	*M* = 44 (35–54)	*M* = 42 (22–55)	*M* = 43 (28–55)	
	*SD* = 7.48	*SD* = 6.22	*SD* = 8.84	*SD* = 8.57	

^a^Two participant’s data were removed for incorrect completion of the measure

^b^Classifications are based on the performance of individuals aged 11–17 years old who have no disabilities (n = 193)

### Questionnaire

For the questionnaire, the participants rated how much each sense affected them specifically in the classroom. These ratings were on a scale from 1 to 10 (1 being the lowest and 10 being the highest). The mean scores were calculated for each sense. Hearing was rated as being the sense that affected the participants the most in the classroom (*M* = 6.18, *SD* = 2.90), followed by touch (*M* = 4.88, *SD* = 2.26), smell (*M* = 4.29, *SD* = 2.98) and vision (*M* = 4.06, *SD* = 2.70).

Data from the questionnaire showed that 88 % of the participants were affected by issues relating to hearing, 75 % by touch, 50 % by vision and 38 % smell (Table 2). All of the participants reported difficulties with at least one sense.

**Table 2:** The participant’s scores from the questionnaire rating how much each sense affects them in the classroom

	Senses			
	Hearing	Touch	Smell	Vision
No. of participants affected by the sense^a^	14	12	7	9
Percentage of participants affected by the sense	88	75	44	56
Means, ranges and standard deviations	*M* = 6.18 (1–9)	*M* = 4.88 (1.5–8)	*M* = 4.29 (1–10)	*M* = 4.06 (1–9)
	*SD* = 2.90	*SD* = 2.26	*SD* = 2.98	*SD* = 2.70

^a^Out of a total of 16 participants

#### Question 1: Do You Think These Sensory Difficulties Affect Your Learning? If Yes, How Do They Do This?

All answers for hearing were ‘yes’, the participants do think their sensory difficulties affect their learning. The majority of responses described a *reduction in concentration*, which would lead to the participants missing sections of their lessons. Noise often provoked a physical response in the participants leading to further distraction. Three comments from the “hearing” questions described the anxiety that is caused by anticipating an unexpected adverse input in this modality, for example, “anxiety every time I did cookery because it was about a certain teacher”. One of the comments under *other* was “when I am in mainstream classrooms I can hear lots of conversation/noise and it makes me feel tired” and is a response that is repeated in vision.

Out of the participants that reported sensory issues with touch, five stated that they did not think these problems affect their learning. *Reduction in concentration* was cited by three participants, but touch issues are not constant for participants. One of these comments came with an explanation: “only when I’m stressed, or things go wrong”. This suggests that touch processing can become an issue when a person is already in a negative state.

Similarly to hearing, *reduction in concentration* has the highest count for vision compared to the other codes in this sense. The code *sometimes*, combined with the same code from touch, provides further evidence of sensory processing not being a constant issue. Very little detail was given by the participants for these comments, and so it is not clear what factors might cause sensory issues to be present. One participant under *other* stated that they get tired; a comment describing being tired was also made for hearing. A total of three participants stated that vision difficulties did not affect their learning.

Out of the few participants who reported smell as causing them difficulties at school the majority stated that they did not consider this to affect their learning. The participants who did consider this sense an issue found it to reduce their concentration. The wording used within both of the comments for this code could suggest that in the same way as vision and touch, these issues are not constant: “sometimes” and “they *can* distract me”.

#### Question 2: How Does it Make You Feel When You Experience These Sensory Difficulties?

 Hearing again was a major concern reported by most of the participants and generated the greatest number of codes. *Anxious* and *uncomfortable* were the codes most commonly found in the questionnaire responses. These were closely followed by *frustrated*, *annoyed* and *physical discomfort*. *Physical discomfort* is made up of descriptions that range from what can be considered a minor issue (“the scraping sound makes my tummy feel strange”) to actual pain (“shouting makes my ears hurt”).

Two codes were identified for touch: *physical discomfort* and *anxious* with the latter receiving the majority of the counts. *Physical discomfort*, similarly to hearing, ranges from a minor issue to actual pain. Another interesting comment within *other* was feeling “even more stressed” when experiencing touch difficulties. In question 1 the same participant reported to only experience sensory difficulties when stressed; touch making them even more stressed shows a stress/sensory vicious cycle.

Three codes are found within vision, all with two counts each: *doesn’t have an effect*, *physical discomfort* and *positive reaction*. Within *other* “annoyed” appears as it does in hearing and “I find it hard to concentrate” links into the *reduction in concentration* code found in all of the senses in question 1. One participant described both a positive and a negative reaction to vision dependent on the situation: “they can make me feel happy, they can make me feel sad, depends on the situation”. *Physical discomfort* ranged from a minor issue to actual pain. Two participants stated that their vision difficulties do not have an effect on them. This is consistent with the findings in question 1 for vision, touch and smell.

The only comment that was consistent enough to become a code for smell was *physical discomfort*. Unlike the other three senses this code only contains comments describing minor physical discomfort.

Overall, one consistent code was found across all four senses: *physical discomfort* which occurred a total of 9 times. This means that having an adverse physical response to sensory input is something that happens in all senses across all participants. Another consistent code found across hearing, touch and vision was *anxiety* and this appeared 11 times.

#### Question 3: Do You Think There are Any Positives About How You Experience Sensory Difficulties?

 For hearing and smell the majority of the participants stated that they did not think there were any positives to how they experienced these sensory difficulties. For touch and vision the number of participants who thought there were positives was the same as the number of participants that thought there were negatives. The only answers that were expanded upon were the *yes* answers. From these, two codes were identified: *the sensory input produces a positive* and *a positive comes as a side effect*.

## Discussion

Similar to past research, all of our participants showed processing difficulties in at least one of the sensory profile quadrants, and 86 % of the participants scored outside the normal range on two or more of the quadrants. The participants were aware of their sensory issues, and all reported difficulties in the classroom within at least one sensory domain. Hearing was rated as being the sense that particularly affected the participants followed by touch, smell and vision. The adolescents in the study reported that sensory sensitivity affected their learning to some extent, and reported their sensory experiences within the classroom as largely negative.

Our findings from the AASP match those reported in previous research (Crane et al. 2009; Myles et al. 2007), and support sensory processing abnormalities as a feature in ASC. The questionnaire developed for this study furthered these results by enabling participants to explain how they experienced sensory processing issues in the classroom. All participants reported difficulties in the classroom within at least one sensory domain, which is consistent with previous research (Dawson and Watling 2000). Sensory issues involving hearing affected the participants most within the classroom, followed by touch, smell and vision. Previous research has found difficulties with auditory processing being reported the most by individuals with ASC outside of the classroom context (Tomchek and Dunn 2007).

In relation to learning, the most consistent theme across all four senses is a *reduction in concentration* with a total of 19 counts. Sensory issues with hearing, touch, vision and smell all distract from the focus of the classroom. Some participants reported problems in all four senses, and this significantly increases the amount of sensory input that is hindering concentration. Reduced concentration is consistent with first-hand reports of sensory processing difficulties in ASC both generally (Carrington and Graham 2001; Jones et al. 2003) and specifically in the classroom (Grandin 2014). Uncomfortable sensory stimuli may focus attention away from key elements of the environment (e.g., a class presentation) and sensitise attentional mechanisms to focus in on the distracting sensory stimuli (Desimone and Duncan 1995): thus environmental stimuli, which may not elicit bottom up attentional mechanism, or only weakly so, in typically developing children may become a prominent focus of attention in children with ASC. In this respect children with ASC may find it difficult to take top down control of competing attentional stimuli due to the negative and sometimes painful effect the stimuli provoke leading to poorer academic performance (Ashburner et al. 2008). Noise within the classroom and hypersensitivity to this noise may also affect children beyond their reported level of a reduction in concentration. For example, Foxe et al. (2015) demonstrated that background noise disrupts multisensory integration in high-functioning individuals with autism making it harder for them to integrate phonemes with lip shapes; a process that aids comprehension in noisy environments.

Not all the participants considered their sensory problems to affect their learning. This means that whilst ASC individuals do experience sensory problems, these do not always have a significant self-perceived negative impact. The only exception to this is with hearing; all participants reported this to affect their learning.

One issue which has not received much attention in the literature is the feelings sensory issues provoke in individuals. Two codes were found consistently across all four senses: *physical discomfort* and *anxiety*. Physical discomfort consists of comments on a range from what could be considered moderate discomfort to physical pain. This level of physical discomfort is consistent with first-hand reports of ASC sensory experiences (e.g., Jones et al. 2003). Pain thresholds in ASC have been reported to be lower than in typically developing controls (Cascio et al. 2008), and these findings support a general over reaction to normally innocuous stimuli; however, evidence in this field is still equivocal (see Moore 2015). Children with ASC may also show greater behavioural reactions to pain than typically developing controls (Nader et al. 2004). An inverse pain response (reporting pain when none should be present) has been documented in clinical settings, and discomfort may often be reported in the absence of confirmation of low sensory thresholds, suggesting that problems are occurring at higher stages of processing rather than being the result of heightened acuity at the sense organs (Tantam 2012). Sensory input to the point of causing such physical discomfort is consistent with the participant’s scores for the sensory sensitive and sensation avoiding quadrants from the AASP. These identified the participants as having a low neurological threshold, which would mean they are over responsive to stimuli and would explain their levels of discomfort. Anxiety produced from sensory sensitivity was another source of discomfort and distress for the participants. Anxiety in children with autism is higher than in children without autism (Vasa et al. 2013) and is likely to come from many different sources, but concentration on sensory issues could prove a relatively easy way to reduce levels of anxiety in the school environment.

Not all aspects relating to sensory issues were reported as being negative. The code *sensory input produces a positive* is found across three senses (touch, vision and smell), and highlights that sensory input can produce positive experiences as well as the negatives ones. Positive aspects of sensory stimuli have also been reported in adults (Robertson and Simmons 2015). The majority of positive comments include examples of sensory experiences providing a bonus, for example “I know what’s going on because my hearing is really good”. There is one comment that stands out as being slightly different to the rest in this code: “you know what bothers you”. This comment is interesting because the participant is not stating a positive ability that their sensory processing provides, instead they are acknowledging they have negative sensory experiences, but knowing this information is in itself a positive. Robertson and Simmons (2015) also point out that being aware of stimuli that cause sensory stress and having control over them is an important method for elevating stress and anxiety.

A consistency that has been found across all the data is that sensory experiences are not a constant state, but are dependent on changing variables, for example sensory modality, specific sensory inputs and the situation a person is in at the time. For example, participants made the following comments: “only when I’m stressed”, “they can make me feel happy, they can make me feel sad, depends on the situation” and “it can make me feel nice, or sick or disgusted, depending on the smell”. These comments support findings by Smith and Sharp (2013) that the impact of sensory stimuli on the individual is mediated by stress and situation. These findings have obvious implications for a one size fits all approach to dealing with sensory stress in children with ASC and place the emphasis on schools to work with each child to produce a sensory stress profile for that child.

### Limitations

The majority of written responses in the current study were short and contained little detail, for example “it hurts”. This may reflect the fact that the researcher was not present whilst the data was collected. The explanations of the research came from staff at the schools, who had close contact with the participants, and it was thought that participants would be more comfortable with this interaction rather than interacting with a stranger. In future research alternative methods could be used to elicit more in depth responses, for example, as in Smith and Sharp (2013), interviews could be conducted using Instant Messaging software.

A limitation of the AASP is that unlike the SP, the senses are combined in order to calculate the quadrant scores. Individuals with ASC and they can have very different experiences depending on the sense (Jones et al. 2003), and they can have different sensory processing styles for different senses (Bartlet 2014; Dunn 2007). The results from the questionnaire in the current study are consistent with this. This means that if a participant scored very low in one of the senses in the AASP it could pull down their overall score and not accurately reflect their sensory experiences. Being able to investigate each sensory domain separately would significantly add to the understanding of sensory processing of individuals with ASC (Tavassoli et al. 2013).

### Future Research

Our research suggests that sensory processing issues share some commonality among the participants in this research, but there is also scope for these experiences to be unique in nature. Stress and environment mediate this experience and can result in stimuli provoking distress or pleasure in the individual. One issue is that the outsider may have little knowledge of the turmoil sensory input may have on individuals with ASC, as intensity may vary depending on a number of factors.

Our future research will extend the work reported here by marrying the experiences reported by participants with physiological measurements of stress. In this way we can monitor real time stress and set thresholds which when passed signal to the participant, they report their experience. Such techniques, using smart watch technology, will provide a richer understanding of the subjective and bodily experience of sensory issues, and could provide a real time early warning system to teachers. Whilst our work has considered sensory processing issues in terms of reaction to common sensory inputs we acknowledge that the classroom situation may be made difficult due to multisensory integration problems (Kwakye et al. 2011). For example, following the lesson or gaging interaction with peers may be rendered difficult; research into both aspects of sensory processing have potential to improve the daily life of school children with ASC.

The participants in the current paper reported hearing to be the sense that was the most problematic in the classroom, which is consistent with the high prevalence of auditory hypersensitivity in comparison to the other senses in individuals with ASC (Gomes et al. 2004). Future research should investigate mediating factors, such as control over the sensory input and aversion to specific inputs, to identify the extent that these affect each sense and why hearing is significantly more affected in ASC.

## Conclusion

The participants in the current study recorded intense sensory processing patterns which could lead to difficulties in the classroom. Sensory issues relating to hearing had a major impact on the participants and vision affected them the least. Content analysis revealed that most of the participants considered their sensory experiences to affect their ability to learn. Consistent difficulties caused by sensory experiences were found with concentration, anxiety and discomfort. School is a significant part of a child’s life and research should continue to explore how sensory difficulties impact a child with ASC’s experiences there. Increased understanding can lead to more appropriate interventions to help children with ASC access the same level of education and schooling experience as neurotypical children.

## Appendix 1: Questionnaire

**Table:** To start with, please describe the ‘typical’ classroom that you use at school. You should mention the size of the room, average number of students and teachers in the room and any details about the environment you think are important

Touch									
These questions are specifically about how touch difficulties affect you in the typical classroom you have described									
On a scale of 1–10 (1 being the lowest and 10 being the highest), how much does touch affect you?									
1	2	3	4	5	6	7	8	9	10
Do you think these touch difficulties affect your learning? If yes, how do they do this?									
How does it make you feel when you experience these touch difficulties?									
Do you think there any positives about how you experience touch differences?									
Vision									
These questions are specifically about how vision difficulties affect you in the typical classroom you have described.									
On a scale of 1–10 (1 being the lowest and 10 being the highest), how much does vision affect you?									
1	2	3	4	5	6	7	8	9	10
Do you think these vision difficulties affect your learning? If yes, how do they do this?									
How does it make you feel when you experience these vision difficulties?									
Do you think there any positives about how you experience vision differences?									
Hearing									
These questions are specifically about how hearing difficulties affect you in the typical classroom you have described									
On a scale of 1–10 (1 being the lowest and 10 being the highest), how much does hearing affect you?									
1	2	3	4	5	6	7	8	9	10
Do you think these hearing difficulties affect your learning? If yes, how do they do this?									
How does it make you feel when you experience these hearing difficulties?									
Do you think there any positives about how you experience hearing differences?									
Smell									
These questions are specifically about how smell difficulties affect you in the typical classroom you have described									
On a scale of 1–10 (1 being the lowest and 10 being the highest), how much does smell affect you?									
1	2	3	4	5	6	7	8	9	10
Do you think these smell difficulties affect your learning? If yes, how do they do this?									
How does it make you feel when you experience these smell difficulties?									
Do you think there any positives about how you experience smell differences?									

## Appendix 2: The Coding Manual

See Tables 3, 4, 5 and 6.

**Table 3:** The codes and code examples for question 1

Code	Code description	Code example
Reduction in concentration	Describes a reduction in concentration or a behaviour that would mean a reduction in concentration	6b Lose concentration
		2c I get distracted
Misses parts of lessons because of a physical response	A physical response to hearing difficulties that would lead to missing parts of a lesson	7b I sometimes miss the explanations of work because I put fingers in my ears
		1b Sometimes I have to leave the room
Unable to hear	Describes an inability to hear	2a Won’t be able to hear the teacher
		1c I often don’t understand or mishear tasks
Creates anxiety about a specific situation	States ‘anxiety’ or ‘worry’ in relation to a specific situation where there is a hearing difficulty	3b Anxiety every time I did cookery because it was about a certain teacher
		4a Only if worrying about a fire drill
Sometimes	States ‘sometimes’ or describes the issue of as not being constant	2c Occasionally they do
		9b Only when I’m stressed, or things go wrong, don’t like being near people
No	States ‘no’ and ‘they don’t’ or describes that it does not affect their learning	1c They don’t
		4b I don’t think it affects my learning a lot
Inability to access the board	Describes an inability to access the board for a purpose	4b It affects me writing information from the smart board
		1c I cannot see the board

**Table 4:** The codes and code examples for question 2

Code	Code description	Code example
Frustrated	States ‘frustrated’ or describes behaviour associated with frustration	2a Frustrated
		2c I get frustrated with the thing that is making the noise
Anxious	Describes a state associated with anxiety	4a Scared
		3b Anxious
Physical discomfort	Describes a physical discomfort	3c The scraping sound makes my tummy feel strange
		3c It hurts
Uncomfortable	States ‘uncomfortable’	1b Uncomfortable
		9b Uncomfortable
Annoyed	States ‘annoyed’	2b Annoyed that other people are distracting me from my learning
		1c Annoyed
Doesn’t have an effect	Describes no effect, everything stays the same	4b It doesn’t affect me
		5b No change, not really a difficulty
Positive reaction	Describes a positive reaction	2b They can make me feel happy, depends on the situation
		2c Sometimes I find the behaviour of the other kids moving around/messing around funny

**Table 5:** The codes and code examples found for question 3

Code	Description	Code examples
No	States ‘No’	3a No
		9b No
Yes	Describes something positive about the sensory differences	2b I think sometimes it can be relaxing and take stress away
		8b I know what’s going on because my hearing is really good
Don’t know	States ‘don’t know’ or ‘not sure’	4a Not sure
		7b Don’t know

**Table 6:** The codes and code examples found within the ‘Yes’ code for question 3

Code	Description	Code examples
The sensory input produces a positive	An example of sensory input being processed and resulting in a positive	2c Sometimes it feels good to give someone a hug
		2b Sometimes the smell is nice and makes me relax
A positive comes as a side effect	A positive does not directly come from the sensory input but instead comes as a side effect of how this is experienced	8b I know what’s going on because my hearing is really good
		1c You know what bothers you

## Appendix 3: Comments that Appear in the Code *Other*

See Tables 7 and 8.

**Table 7:** The comments found in the code ‘other’ for question 1 for hearing, touch, vision and smell

Code	Description	The comments for each sense			
		Hearing	Touch	Vision	Smell
Other	Only appears once and does not fit into an existing code	4a Sometimes people whisper and I don’t know what they are talking about	1a It makes me cross	7b I get tired	1a Yes, if it’s a bad smell
		8b When I am in mainstream classrooms I can hear lots of conversation/noise and it makes me feel tired	2c Fuzzy

**Table 8:** The comments found in the code ‘other’ for question 2 for hearing, touch, vision and smell

Code	Description	The comments for each sense			
		Hearing	Touch	Vision	Smell
Other	Only appears once and does not fit into an existing code	3c NO	5b Why is this person here?	4a I find it hard to concentrate	2b It can make me feel nice, depending on what the smell is
		7b Don’t know	5b Where has their hand been?	2b They make me feel sad, depends on the situation	2a Wish it would go away
		3a I feel like huffing and puffing	2c I’m scared that I might barge into someone or step on their foot and then they will tell on me and I will get into trouble	1c Annoyed	4a Not too worried
			1a Cross		3b Get used to it in a little while
			3b Annoyed		
			9b Even more stressed		
			2c I feel very agitated		
			2b Uncomfortable		
			2a Am okay, doesn’t make me feel bad		
